# Ultrastructural Evaluation (SEM) of *Ascaris lumbricoides* Eggs Treated with Silver Nanoparticles Biosynthesised by *Duddingtonia flagrans* Using Scanning Electron Microscopy (SEM)

**DOI:** 10.3390/pathogens15010095

**Published:** 2026-01-15

**Authors:** Carolina Magri Ferraz, João Pedro Barbosa de Assis, Eduarda Cavalini Guerini, Juliany Veloso Leal, Filippe Elias de Freitas Soares, Marcio Fronza, Jackson Victor de Araujo, Luís Madeira de Carvalho, Fabio Ribeiro Braga

**Affiliations:** 1 Laboratório de Parasitologia Experimental e Controle Biológico, Universidade Vila Velha, Rua São João, 48, Vila Velha 29102-920, Brazil; carolina.ferraz@uvv.br (C.M.F.); jpbarbosa-deassis@hotmail.com (J.P.B.d.A.); eduarda.cavalini@hotmail.com (E.C.G.); julianyveloso4@gmail.com (J.V.L.); 2 Laboratório de Bioquímica, Universidade Federal de Lavras, Rua Professor Edmir Sá Santos, Lavras 37203-202, Brazil; filippe.soares@ufla.br; 3 Laboratório de Produtos Naturais, Universidade Vila Velha, Rua São João, 48, Vila Velha 29102-920, Brazil; marcio.fronza@uvv.br; 4 Programa de Pós-Graduação em Medicina Veterinária, Universidade Federal Fluminense, Avenida Almirante Ary Parreiras, 507, Niterói 24230-321, Brazil; jvictor@ufv.br; 5 Department of Animal Health, Faculty of Veterinary Medicine, University of Lisbon, Avenida Universidade Técnica, 1300-477 Lisboa, Portugal; madeiradecarvalho@fmv.ulisboa.pt

**Keywords:** biological control, nematophagous fungi, ovicidal activity, soil-transmitted helminths, biogenic nanoparticles

## Abstract

*Ascaris lumbricoides* is one of the most epidemiologically significant soil-transmitted helminths, and the environmental persistence of its eggs is largely attributed to their robust structural architecture. The search for ovicidal alternatives capable of overcoming this barrier has increasingly focused on metallic nanoparticles obtained through biological synthesis. Scanning electron microscopy (SEM) was employed to evaluate the ultrastructural effects of silver nanoparticles (AgNPs) biosynthesised by the nematophagous fungus *Duddingtonia flagrans* on *A. lumbricoides* eggs. Ultraviolet-visible spectroscopy and transmission electron microscopy confirmed the synthesis of AgNPs, revealing predominantly spherical, well-dispersed particles with an average diameter of 9.22 ± 4.9 nm. Cytotoxicity assays indicated an IC_50_ of 7.7 µg/mL. SEM analyses showed that eggs in the control group maintained intact morphology, with no apparent deformities. In contrast, exposure to AgNPs induced pronounced structural alterations, including marked wrinkling, surface erosion and shell collapse, suggesting disruption of multiple layers. Albendazole alone produced deep linear fissures consistent with internal metabolic failure, though with minimal external erosion. The combined treatment with AgNPs and albendazole resulted in severe degradation. These findings demonstrate that AgNPs exhibit significant ovicidal activity and may serve as effective adjuvants to enhance the action of conventional anthelmintics against highly resistant helminth eggs.

## 1. Introduction

Soil-transmitted helminthiases (STHs) are classified among the most prevalent parasitic infections worldwide [1,2]. It is estimated that approximately 1.5 billion people are affected, especially preschool-aged children and populations exposed to poor sanitary and environmental conditions [3,4]. STHs also impose a substantial socioeconomic burden, with global losses estimated at roughly US$ 139 billion per year [5]. Within the group of STHs, *Ascaris lumbricoides*, the etiological agent of ascariasis, is one of the most prevalent species. Infection occurs by means of the ingestion of embryonated eggs present in soil, food, and contaminated water [6]. This helminth is also notable for its antiquity, with records documented in civilizations such as Mesopotamia and Greece [7].

*Ascaris lumbricoides* life cycle depends critically on external factors, requiring moist and shaded environmental conditions for eggs to develop to the infective stage. The extreme resistance of *A. lumbricoides* eggs is widely attributed to their complex ultrastructure and low shell permeability, a typical feature of geohelminth eggs [8]. In systematic evaluations, Lysek et al. [8] described the three fundamental structural layers of the eggshell, including their organization, thickness, density, and surface continuity. Those authors proposed that each layer provides distinct physiological functions, such as mechanical resistance, osmotic isolation, and reduced permeability to physical and chemical agents, ensuring prolonged egg survival in the environment. In further observations, the authors suggested that the inner lipid layer, approximately 1 µm thick, undulated, and highly impermeable is responsible for the physicochemical resistance of the egg. In agreement with these findings, the need to identify effective strategies for controlling STH eggs persists [9].

Albendazole is frequently used in mass drug administration (MDA) campaigns as the primary tool for controlling intestinal helminth infections. However, discrepancies exist regarding the ovicidal activity of certain benzimidazoles, highlighting the need for advancements in alternative control measures targeting eggs, which function as sources of infection [10,11].

In this context, the need to combat helminth eggs and larvae harmful to One Health, such as *A. lumbricoides* eggs in the environment, has driven decades of biotechnological research [12]. This is due to the integrated impact that STHs exert on human, animal, and environmental health, as *A. lumbricoides* eggs present in the environment represent persistent sources of infection, directly affecting human populations, contaminating soil and water, and contributing to zoonotic and environmental transmission cycles. Historically, Lysek and Sterba [13], in pioneering work, identified a critical vulnerability in the previously considered impenetrable eggshell of *A. lumbricoides* using the fungal species *Verticillium chlamydosporium*. Decades later, Braga et al. [14] demonstrated the in vitro efficacy of nematophagous fungi (environmental biological control agents) in colonizing, penetrating, and subsequently destroying these eggs.

Building on these findings, recent research has explored the use of silver nanoparticles biosynthesized by the nematophagous fungus *Duddingtonia flagrans* for the in vitro control of nematode eggs and larvae, with promising results [15,16,17]. However, unlike previous studies that focused on nematicidal, larvicidal, and ovicidal activity, the present study provides the first ultrastructural characterization, by scanning electron microscopy, of the damage induced specifically in *A. lumbricoides* eggs. In addition, this work presents a comparative morphological evaluation of eggs exposed to AgNPs alone, albendazole alone, and their combined application. Therefore, the present study aimed to evaluate the effects of silver nanoparticles derived from *D. flagrans* on the morphology of *A. lumbricoides* eggs.

## 2. Materials and Methods

### 2.1. Biosynthesis and Confirmation of Silver Nanoparticles Derived from Duddingtonia flagrans

Mycelial discs (≈5 mm in diameter) of *D. flagrans* (isolate AC001), previously cultured on Potato Dextrose Agar (PDA; Kasvi^®^, São José dos Pinhais, PR, Brazil), were transferred to Erlenmeyer flasks containing 200 mL of Potato Dextrose Broth (PDB; Kasvi^®^, Madrid, Spain). The flasks were incubated in a refrigerated orbital shaker (SL-223, Solab^®^, Piracicaba, SP, Brazil) for 15 days at 120 rpm and 25 °C.

After incubation, the fungal biomass was separated by filtration using a sterile sieve and washed with sterile distilled water. Subsequently, 10 g of the biomass were transferred to Erlenmeyer flasks containing 100 mL of distilled water and 1 g of chitosan (Êxodo Científica^®^, Sumaré, SP, Brazil) to produce the fungal filtrate. These flasks were incubated under orbital shaking (120 rpm, 25 °C) for 15 days. At the end of this period, the material underwent two filtration steps: first through sterile paper filters (20 μm), and then through syringe filters (0.22 μm), yielding a sterile, cell-free fungal filtrate, as described by Costa Silva et al. [18].

For nanoparticle biosynthesis, a 1 mM silver nitrate solution (AgNO_3_; Êxodo Científica^®^, Brazil) was used. The fungal filtrate and AgNO_3_ solution were mixed at a 1:50 ratio in Erlenmeyer flasks and incubated in an orbital shaker (120 rpm) at 60 °C in the dark for 72 h. After this incubation period, the biosynthesis of AgNPs-*D. flagrans* was initially confirmed by UV–Vis spectroscopy using a UV-M51 spectrophotometer (BEL Engineering^®^, Monza, Italy). Readings were performed with a 1 nm resolution in a continuous scan from 200 to 1000 nm, as described by Elamawi et al. [19], and analyzed using the VISME online platform. As controls, samples of the fungal filtrate and the 1 mM AgNO_3_ solution were analyzed separately. Nanoparticle formation was further confirmed by transmission electron microscopy (TEM), which enabled the assessment of morphology, distribution, and size of the biosynthesized AgNPs [18]. TEM images were analyzed using the ImageJ software (version 1.54k, English language; National Institutes of Health, Bethesda, MD, USA) [20].

### 2.2. In Vitro Cytotoxicity Assay of Silver Nanoparticles Derived from Duddingtonia flagrans

The L929 fibroblast cell line (ATCC^®^ CCL-1™) was used for the cytotoxicity assessment. Cells were cultured in Dulbecco’s Modified Eagle’s Medium (DMEM; Gibco-BRL Life Biotechnologies^®^, Grand Island, NY, USA), supplemented with 10% fetal bovine serum, 100 IU/mL penicillin, and 100 µg/mL streptomycin. Cultures were maintained at 37 °C in a humidified atmosphere containing 5% CO_2_.

Cytotoxicity was evaluated using the standard MTT colorimetric assay [21]. Briefly, 100 μL of the L929 cell suspension (3 × 10^4^ cells/mL) were seeded into 96-well plates. After overnight incubation at 37 °C with 5% CO_2_, the cells were exposed to AgNP concentrations ranging from 1.5 to 100 μg/mL. Following 24 h of incubation, the supernatant was removed and 100 μL of MTT solution (3-(4,5-dimethylthiazol-2-yl)-2,5-diphenyl tetrazolium bromide; 1 mg/mL) were added to each well and incubated for an additional 2 h. The MTT solution was then carefully aspirated and DMSO was added, followed by 5 min of orbital agitation. The MTT assay was conducted according to the classical protocol described by Mosmann [21], with absorbance measured at 570 nm, a wavelength widely used and validated for quantifying solubilized formazan crystals. A single-wavelength reading was adopted because no relevant optical interference from the medium or nanoparticles was observed under the experimental conditions employed. All assays were performed in triplicate.

### 2.3. Determination of Silver Nanoparticles Concentration

The mass concentration of biosynthesized AgNPs was determined using a gravimetric method. After biosynthesis, 2 mL aliquots of the AgNP suspension were transferred to microcentrifuge tubes and centrifuged at 12,000 rpm for 20 min. The supernatant was carefully removed, and the resulting nanoparticle pellets were dried in an incubator at 37 °C for 24 h [18]. The dry AgNP powder was weighed, and the measured mass was used to calculate the nanoparticle concentration. The AgNPs were then resuspended in sterile distilled water to obtain the desired working concentrations, including the IC_50_ value (7.7 µg/mL).

### 2.4. Collection of Ascaris lumbricoides Eggs

*Ascaris lumbricoides* eggs were obtained by dissecting adult female specimens. The eggs were examined under light microscopy to evaluate their structural integrity. Viability criteria followed modified methodologies described by Ferraz et al. [17] and Cáceres et al. [22]. The following parameter was assessed: shell integrity, defined as the presence of intact structures with continuous coverings, or, conversely, contraction, rupture, and loss of membrane continuity.

### 2.5. Experimental Assay

The experimental assay was conducted in sterile 2 mL microtubes. The treated group received the concentration of AgNPs-*D. flagrans* corresponding to the IC_50_ value obtained in the MTT assay (7.7 µg/mL). The control drug group received albendazole. The albendazole concentration used in this study (5.12 µg/mL) corresponds to the IC_50_ previously determined in L929 fibroblast cells, as reported by Oroojalian et al. [23]. This concentration was adopted as a reference biological dose for comparative purposes and to evaluate its effects on parasite eggs under in vitro conditions. Albendazole was prepared by direct dissolution in sterile distilled water at a final concentration of 5.12 µg/mL, without the use of organic solvents. All concentrations were standardized after formation of the experimental groups. The groups were organized as follows: Group 1: 200 *A. lumbricoides* eggs + 1 mL sterile distilled water; Group 2: 200 *A. lumbricoides* eggs + 1 mL AgNPs-*D. flagrans*; Group 3: 200 *A. lumbricoides* eggs + 1 mL albendazole; Group 4: 200 *A. lumbricoides* eggs + 1 mL AgNPs-*D. flagrans* + 1 mL albendazole. All treatments were performed in triplicate.

Microtubes were maintained at room temperature (25 °C ± 2 °C), protected from light, for seven days. At the end of the incubation period, samples were processed for analysis under scanning electron microscopy (SEM) at the Carlos Alberto Redins Ultrastructural Analysis Laboratory (LUCCAR), Federal University of Espírito Santo (UFES).

### 2.6. Scanning Electron Microscopy (SEM) Sample Preparation

For ultrastructural analysis, *A. lumbricoides* eggs from all experimental and control groups were processed simultaneously under identical conditions. Samples were initially washed in phosphate-buffered saline (PBS) and fixed in 0.1 M cacodylate buffer for 24 h. Subsequently, specimens were post-fixed in a solution containing 1% osmium tetroxide (OsO_4_) in 0.1 M cacodylate buffer supplemented with 1.25% potassium ferrocyanide. After post-fixation, samples were washed in 0.1 M cacodylate buffer followed by ultrapure water. Dehydration was performed using a graded ethanol series (30%, 50%, 70%, 90%, and 100%). The dehydrated material was dried by the critical point method using liquid CO_2_ as the transitional fluid and subsequently air-dried at 25 °C for 15 min. The dried specimens were mounted on metal stubs according to the required orientation and sputter-coated with a thin layer of gold using an ion sputter coater (JFC-1100; JEOL). The samples were examined under a scanning electron microscope (LEO 435 VP 501B, Philips, Eindhoven, The Netherlands), with electron acceleration voltages ranging from 10 to 20 kV.

## 3. Results and Discussion

### 3.1. Ultraviolet-Visible Spectrophotometric and Transmission Electron Microscopy Analyses of Silver Nanoparticles Derived from Duddingtonia flagrans

**Figure 1:**
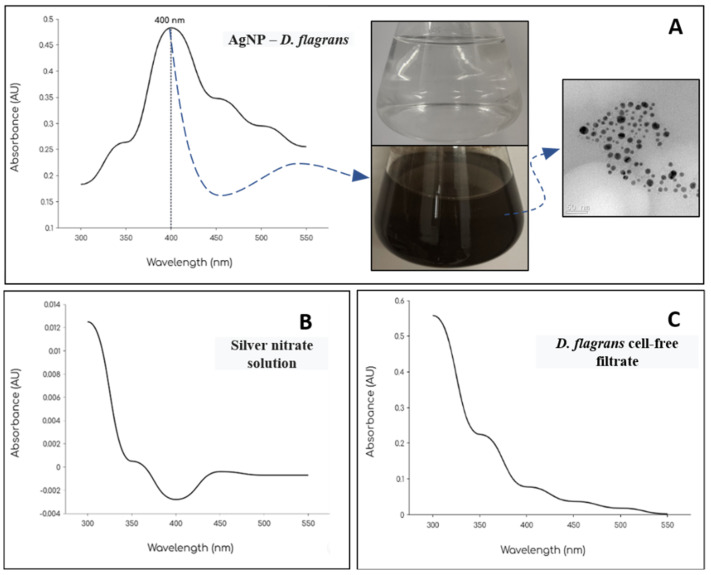
UV-Vis absorption spectrum of: (**A**) AgNPs-*Duddingtonia flagrans*, (**B**) 1 mM silver nitrate solution, and (**C**) cell-free fungal filtrate. The images in the upper right corner (**A**) show the visual difference in the fungal filtrate + AgNO_3_ solution over the incubation period and the image of AgNPs-*D. flagrans* obtained by TEM.

The formation of AgNPs-*D. flagrans* was confirmed by ultraviolet–visible (UV–Vis) absorption spectroscopy following the visual color change in the reaction mixture from colorless to brown, indicative of the reduction of Ag^+^ ions to metallic silver (Figure 1). This visual shift is attributed to the action of metabolites present in the fungal filtrate, which function as reducing agents [24]. According to Guilger-Casagrande and de Lima [25], fungi possess an intrinsic capacity to secrete large quantities of proteins and enzymes capable of reducing silver ions into nanoparticles, while simultaneously encapsulating and stabilizing the particles to prevent aggregation.

The UV–Vis spectrum of the AgNP-containing sample displayed a well-defined absorbance peak around 400 nm, consistent with localized surface plasmon resonance (LSPR), a phenomenon characteristic of metallic nanoparticles [24,26]. LSPR occurs when free electrons on the nanoparticle surface resonate with the oscillating electric field of incident light, resulting in strong absorption at specific wavelengths and imparting a distinctive coloration to the colloidal suspension [27].

The optical properties of AgNPs including the peak position and intensity in the UV–Vis spectrum are directly influenced by nanoparticle size, shape, and degree of dispersion. In general, smaller particles tend to produce sharper and more intense peaks [28]. As reported by Alzoubi et al. [26], an absorbance signal centered near 400 nm is typically associated with spherical silver nanoparticles. The AgNO_3_ solution (1 mM) and the cell-free fungal filtrate were analyzed separately as controls; neither exhibited significant absorbance at ~400 nm, confirming that nanoparticle formation occurred only when both the filtrate and silver nitrate were present (Figure 1).

Transmission electron microscopy (TEM) images were used to determine the size of the biosynthesized AgNPs. A total of 300 particles were measured using ImageJ, yielding an average diameter of 9.22 ± 4.9 nm, with particle sizes ranging from 1.6 to 24.5 nm. TEM micrographs revealed predominantly spherical, well-dispersed nanoparticles, with no evident signs of significant aggregation, features commonly associated with biologically synthesized AgNPs (Figure 1). Notably, smaller nanoparticles exhibit larger surface area–to–volume ratios, which may enhance silver ion release and cellular interactions [29].

### 3.2. In Vitro Cytotoxicity Assay

The cytotoxic effects of AgNPs-*D. flagrans* on the L929 fibroblast cell line were evaluated using the MTT colorimetric assay. The AgNPs displayed an IC_50_ value of 7.7 ± 0.5 µg/mL. The IC_50_ value was used in this study as a biologically active yet sublethal reference concentration, commonly adopted in preliminary nanotoxicological and antiparasitic investigations. Numerous studies report that the IC_50_ of AgNPs varies according to particle size, shape, surface coating, and synthesis method [30]. In the present study, TEM revealed that the biosynthesized AgNPs were small and relatively monodisperse, consistent with the findings of Nieves et al. [31], who demonstrated in animal models that smaller AgNPs tend to be more toxic than larger ones due to their increased surface area and enhanced ability to penetrate biological membranes. Furthermore, due to their small size, silver nanoparticles may induce the production of reactive oxygen species (ROS) and/or increase silver bioavailability, contributing to their potent biological activity [32].

### 3.3. Ultrastructural Analysis of Ascaris lumbricoides Eggs

As widely recognized in the literature, the control of *A. lumbricoides* is challenged by environmental contamination and the exceptional resistance of its eggs [13]. This resilience underscores the need for control measures that target the environmental stages of the parasite, thereby justifying the search for biological agents [9,33]. The pioneering work of Lysek and Sterba [13] with *Verticillium chlamydosporium* highlighted the potential of nematophagous fungi an approach now extended to nanotechnology through *D. flagrans*-derived nanoparticles.

**Figure 2:**
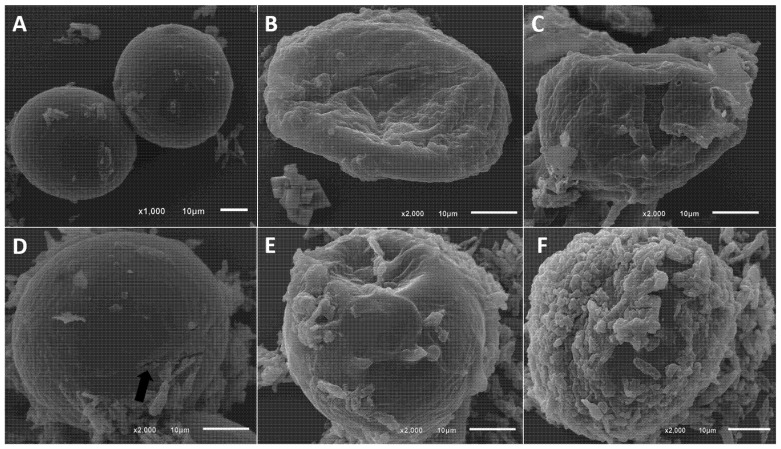
Ultrastructural analysis of *Ascaris lumbricoides* eggs. (**A**) Intact *A. lumbricoides* egg (control group), showing preserved morphology and structural integrity. (**B**,**C**) *A. lumbricoides* egg with evident destruction (group treated with AgNPs-*D. flagrans*). (**D**) *A. lumbricoides* egg (Albendazole-treated group) exhibiting a shell fissure (black arrow). (**E**,**F**) *A. lumbricoides* egg after combined treatment with AgNPs-*D. flagrans* + Albendazole, showing marked rupture and degradation of the eggshell.

In the control group, SEM analysis (Figure 2A) showed the characteristic, preserved morphology of *A. lumbricoides* eggs. The eggs displayed the expected spherical to subspherical shape with no evidence of collapse, depression, or structural deformation [34]. The outer shell exhibited a smooth, continuous surface with a fine, uniform texture, lacking deep rugosities, fissures, fractures, or erosions observed in treated groups. No significant adhered material or residues were detected, indicating the absence of external agents capable of causing damage.

In Group 2 (eggs + AgNPs), SEM revealed marked alterations in eggshell integrity (Figure 2B,C). Treated eggs exhibited extensive structural collapse, loss of sphericity, and pronounced wrinkling of the eggshell surface. Ultrastructural alterations were consistently observed across multiple examined fields in eggs exposed to the AgNP-*D. flagrans*, whereas control eggs maintained intact surface morphology.

In Group 3, treatment with albendazole (ABZ) (Figure 2D) produced linear fissures associated with internal metabolic collapse. ABZ acts internally by disrupting β-tubulin polymerization, impairing energy metabolism and cellular structure in the developing embryo. Although the thick eggshell of *A. lumbricoides* is highly resistant to penetration, once the drug is absorbed, it induces lysis and collapse of the internal embryonic content [13]. This internal collapse generates negative pressure or volume retraction, which manifests externally as deep linear fissures in the otherwise resistant shell. The shell was not disintegrated by surface erosion, as observed with nanoparticles. Importantly, these fissures demonstrate that even when ABZ effectively induces cellular death, the egg relies on its robust shell to withstand structural failure.

In Group 4 (eggs + AgNPs + ABZ), SEM revealed extensive structural damage (Figure 2E,F), characterized by pronounced deformation, collapse, and marked surface disorganization. Compared to the individual treatments, the combined exposure resulted in more extensive ultrastructural alterations of the eggshell. These qualitative observations indicate that the association of AgNPs with albendazole is associated with more pronounced morphological damage under the experimental conditions evaluated. Such findings highlight the potential of nematophagous fungi and their derived nanomaterials as components of integrated control approaches, although further quantitative and functional studies are required to clarify the nature of interactions between AgNPs and conventional anthelmintics.

Ferraz et al. [17] previously reported the first evidence of AgNPs-*D. flagrans* acting on *Toxocara canis* eggs, a zoonotic ascarid of dogs and cats. The present study further demonstrates their deleterious effects on *A. lumbricoides* eggs. Given the extreme resilience of soil-transmitted helminth (STH) eggs, the pursuit of ovicidal agents, such as biosynthesized silver nanoparticles, is justified and increasingly necessary.

Silver nanoparticles possess well-established mechanisms of antimicrobial action. Their small size and high surface area enhance the release of silver ions, which interact with cytoplasmic membranes, nucleic acids, respiratory chain enzymes, and membrane permeability, inducing apoptosis [35]. These same principles may explain their effects on helminth eggs, despite the complexity of eggshell resistance. Evidence indicates that AgNPs can adhere to the eggshell surface (Figure 2B), induce physicochemical alterations, create micro-damage, or modify shell permeability, potentially disrupting embryonic development. However, studies specifically assessing nanoparticle effects on helminth eggs remain scarce, particularly regarding shell integrity, embryogenesis, and post-exposure viability, emphasizing the need for further research. Thus, AgNPs represent a potentially valuable component for future anthelmintic formulations and environmental biocontrol strategies.

In contrast, albendazole (ABZ) primarily targets internal structures in adult and larval stages of parasites. Its ovicidal activity is limited due to the rigid chitin–protein and thick lipid layers of the *A. lumbricoides* eggshell, which restrict drug penetration. Clinically, ABZ is highly effective against motile stages but demonstrates variable and often incomplete efficacy against eggs [36,37,38,39,40]. Sisay et al. [10] also note variability in ABZ efficacy linked to environmental contamination, the high resilience of STH eggs, population age, pharmaceutical brand, and host immune factors. Thus, the primary challenge remains environmental contamination with infective eggs. Research targeting environmental control of pre-parasitic stages (eggs and larvae) may therefore be critical for One Health initiatives [41].

Despite the promising ovicidal and ultrastructural effects observed, potential toxicity associated with silver nanoparticles must also be considered. AgNP toxicity is known to depend on particle size, surface characteristics, concentration, and exposure route. Smaller nanoparticles, such as those evaluated in this study, may enhance biological activity due to increased surface area and silver ion release. However, toxicity profiles differ substantially between in vitro systems, environmental exposure, and systemic administration. In this context, the concentration employed herein was selected as a sublethal reference, and further studies are required to evaluate long-term environmental safety and host toxicity.

Overall, this study demonstrates that silver nanoparticles biosynthesized by *D. flagrans* exhibit promising activity against ascarid eggs, targeting the structural integrity of this highly resistant geohelminth stage. SEM analyses revealed that exposure to the AgNP-containing formulation induced marked ultrastructural alterations, including eggshell deformation, surface erosion, and structural collapse. When combined with albendazole, more pronounced morphological alterations were observed, suggesting an enhanced effect of the combined treatment on eggshell integrity.

These findings support the potential development of integrated strategies that combine environmental biocontrol with optimized pharmacological approaches, employing fungal-based nanotechnology to compromise eggshell defenses and potentially reduce environmental sources of infection. However, it is important to note that the ultrastructural alterations observed by SEM reflect morphological damage to the eggshell but do not directly demonstrate loss of egg viability. Functional assays, such as larval development or hatching tests, would be necessary to confirm whether the observed structural damage effectively prevents embryonation or larval emergence. Therefore, the present findings are interpreted as evidence of eggshell structural compromise, and further studies are required to establish the functional consequences of these alterations.

In addition, although the present findings demonstrate consistent ultrastructural alterations in eggs exposed to the biosynthesized AgNP formulation, some limitations should be considered. The inclusion of additional control groups, such as fungal filtrate with chitosan alone or AgNO_3_ solution alone, could provide further insight into the individual contributions of these components. Accordingly, the observed effects are interpreted in the context of exposure to the AgNP-containing formulation, and future studies incorporating these controls will be valuable for a more detailed mechanistic understanding.

## Data Availability

The original contributions presented in this study are included in the article. Further inquiries can be directed to the corresponding author.
